# Fine Mapping of a Gene (*ER4.1*) that Causes Epidermal Reticulation of Tomato Fruit and Characterization of the Associated Transcriptome

**DOI:** 10.3389/fpls.2017.01254

**Published:** 2017-07-26

**Authors:** Lipeng Cui, Zhengkun Qiu, Zhirong Wang, Jianchang Gao, Yanmei Guo, Zejun Huang, Yongchen Du, Xiaoxuan Wang

**Affiliations:** ^1^Key Laboratory of Biology and Genetic Improvement of Horticultural Crops of the Ministry of Agriculture, Institute of Vegetables and Flowers, Chinese Academy of Agricultural Sciences Beijing, China; ^2^Department of Vegetable Science, College of Horticulture, South China Agricultural University Guangzhou, China

**Keywords:** tomato fruit, epidermal reticulation, water loss, *ER4.1*, cuticle, RNA-seq

## Abstract

The hydrophobic cuticle that covers the surface of tomato (*Solanum lycopersicum*) fruit plays key roles in development and protection against biotic and abiotic stresses, including water loss, mechanical damage, UV radiation, pathogens, and pests. However, many details of the genes and regulatory mechanisms involved in cuticle biosynthesis in fleshy fruits are not well understood. In this study, we describe a novel tomato fruit phenotype, characterized by epidermal reticulation (ER) of green fruit and a higher water loss rate than wild type (WT) fruit. The ER phenotype is controlled by a single gene, *ER4.1*, derived from an introgressed chromosomal segment from the wild tomato species *S. pennellii* (LA0716). We performed fine mapping of the single dominant gene to an ~300 kb region and identified *Solyc04g082540, Solyc04g082950, Solyc04g082630*, and *Solyc04g082910*as potential candidate genes for the *ER4.1* locus, based on comparative RNA-seq analysis of ER and WT fruit peels. In addition, the transcriptome analysis revealed that the expression levels of genes involved in cutin, wax and flavonoid biosynthesis were altered in the ER fruit compared with WT. This study provides new insights into the regulatory mechanisms and metabolism of the fruit cuticle.

## Introduction

Land plants evolved from aquatic ancestors ~450 million years ago (Graham, 1993). In order to survive in dry terrestrial environments, plants evolved a hydrophobic cuticle to cover their aerial surfaces, thereby protecting the interior tissues from desiccation and minimizing non-stomatal water loss (Mccourt et al., 2004; Bargel et al., 2006). In addition, the cuticle plays major roles in protection against mechanical damage, UV radiation and other biotic stresses, such as pathogens and pests (Mccourt et al., 2004; Bargel et al., 2006). The cuticle is composed of a polyester matrix, cutin, which acts as a macromolecular scaffold and within which are embedded a range of intracuticular waxes. Epicuticular waxes are also often deposited on the outer cuticle surface (Kunst and Samuels, 2003; Pollard et al., 2008; Samuels et al., 2008; Yeats and Rose, 2013).

Cutin represents a major structural component of the cuticle, and is composed of covalently cross-linked C16 or C18 oxygenated fatty acids and glycerol (Pollard et al., 2008; Samuels et al., 2008; Beisson et al., 2012; Yeats and Rose, 2013). The cutin matrix is considered to be primarily responsible for the biomechanical properties of the cuticle (Kolattukudy, 1980). In the cuticles of tomato (*Solanum lycopersicum*) fruit, which have been used as a model system for studying cuticle structures and functions (Martin and Rose, 2014), the main cutin monomers are C16-9/10, 16-dihydroxy fatty acids (DiHFA) (Mintz-Oron et al., 2008). The DiHFA monomers are synthesized via a series of enzymatic steps in the epidermis, catalyzed by enzymes such as long-chain acyl-CoA synthetases (LACSs), cytochrome P450-dependent fatty acid oxidases (CYP86A and CYP77A) and glycerol-3-phosphate acyltransferases (GPATs; Li-Beisson et al., 2013). After transport to the apoplast, the cutin monomers are polymerized to form the cuticle matrix (Samuels et al., 2005). Recent studies have identified ATP-binding cassette (ABC) transporters and a cutin synthase (GDSL-motif lipase/hydrolase [GDSL]) that contribute to extracellular transport and polymerization of cutin, respectively (Kurdyukov et al., 2006a; Girard et al., 2012; Yeats et al., 2012, 2014).

Waxes comprise aromatic and aliphatic compounds, including complex mixtures of very long chain fatty acids (VLCFAs) and their derivatives, such as alkanes, alcohols, alkenes, aldehydes, and ketones, as well as terpenoids and sterols (Bernard and Joubès, 2013). Many of the *Arabidopsis thaliana* genes that encode enzymes involved in cuticular wax biosynthesis and transport, or that regulate wax deposition have been well characterized. These include *FATB* (fatty acyl-ACP thioesterase), *LACS* (long chain acylcoenzyme A synthetase), *KCS* (β-ketoacyl-CoA synthase), *KCR* (β-ketoacyl-CoA reductase), *HCD* (3-hydroxyacyl-CoA dehydratase), *ECR* (trans-2,3-enoyl-CoA reductase), *CER1* (ECERIFERUM1), and *WAX2*/*CER3, WSD1* (diacylglycerol acyltransferase 1), *ABCG* (ATP binding cassette ABC transporter) and *LTPG* (glycosylphosphatidylinositol-anchored LTP) (Isaacson et al., 2009; Wang et al., 2011; Bernard and Joubès, 2013; Smirnova et al., 2013). In tomato, the most abundant cuticular waxes in all organs are alkanes; however, branched alkanes, alkenes, and cyclic compounds are also found in tomato leaves, fruits, and anthers (Isaacson et al., 2009; Wang et al., 2011; Smirnova et al., 2013). Previous studies have suggested that the transpiration barrier is mainly provided by the cuticular waxes, with cutin making a less significant contribution (Mintz-Oron et al., 2008).

The cuticle also contains a small amount of polysaccharides and phenolic compounds, such as flavonoids. A recent study demonstrated that polysaccharides also contribute to the mechanical behavior of the cuticle; specifically to the elastic modulus and stiffness (López-Casado et al., 2007). Flavonoids contribute to the elastic properties of the cuticle, adding to the elasticity provided by the polysaccharide fraction (Broun, 2005). Flavonoids are involved in a wide array of plant growth and development processes, including coloration, pathogen resistance, and protection against UV radiation (Broun, 2005; Domínguez et al., 2009). In tomato fruit, flavonoids are localized predominantly in the peel, and the most abundant flavonoid is naringenin chalcone (NarCh), which contributes a yellow/orange color to the fruit, and which acts as a precursor for the biosynthesis of flavonols (Adato et al., 2009). In addition, trace amounts of the flavonol quercetin-3-rutinoside (rutin) accumulate in the peel of ripening tomato fruit (Ballester et al., 2010).

Compared with the cuticles of many model plant species, such as *A. thaliana*, the cuticle of tomato fruit is relatively thick and easy to isolate, as well as being astomatous (Vogg et al., 2004). It provides structural support to maintain fruit integrity has an important role in preventing dehydration of the fruit during development and rapid expansion. It is therefore critical that the cuticle has sufficient elasticity and strength to accommodate fruit expansion (Hen-Avivi et al., 2014). In the absence of such flexibility, fractures in the fruit cuticle can occur, disrupting the hydrophobic barrier. Indeed, in a horticultural context, fruit cracking can be a significant problem, leading to quality loss, poor outward appearance, shelf life reduction, and fungal infection, which in turn results in serious economic losses (Saladié et al., 2007; Isaacson et al., 2009). Peet (1992) classified cracks into the subcategories of radial, longitudinal, concentric cracks, and fractures, and concluded that there are seven main reasons for cracking in fruit, including environmental and genetic factors. However, although many researchers have described the phenomenon of fruit cracking, much remains to be learnt about underlying physiology and genetics and further investigation of the regulation of cuticle biosynthesis and properties is needed.

In this current study, we characterized a tomato genotype with fruit that are characterized by epidermal reticulation (ER) at the mature green fruit stage. The ER fruit showed cuticle fractures and this phenotype was shown to be controlled by a single, dominant gene, derived from the introgression of a chromosomal region from a wild tomato species, *S. pennellii* (LA0716). We analyzed the transcriptome changes that occurred in the ER fruit peel and present insights into cuticle biosynthesis associated with the ER phenotype, thereby extending the current knowledge of fruit surface biology.

## Methods

### Plant material and growth

Seeds of the wild tomato species *S. pennellii* LA0716 were obtained from the Tomato Genetics Resource Center (TGRC; http://tgrc.ucdavis.edu/). *S. lycopersicum* 1052 is an inbred line generated by our group. N72-5 and N72-9 are near-isogenic lines (NILs) in the 1052 background, harboring the *ER4.1* locus from *S. pennellii* LA0716, constructed by our group via marker-assisted selection following the procedures described by Eshed and Zamir (1994), at the Institute of Vegetables and Flowers, Chinese Academy of Agricultural Sciences (CAAS) Beijing.

All tomato plant seedlings were grown in 32-plug trays and cultivated in the same greenhouse in the farm of the Institute of Vegetables and Flowers, CAAS (Beijing, China). Fully opened flowers were manually pollinated and tagged in order to stage the fruit. The ER phenotype on the fruit skin was visible to the eye at the mature green stage fruit (~30 DAP; days after pollination).

### Histological morphology of fruit pericarp tissue

Fruit samples, including the cuticle, were fixed in ethanol-acetic acid (3:1, v/v) at room temperature. The samples were placed in 70% ethanol, dehydrated using a graded ethanol series, followed by a xylene/ethanol series replacement step, and embedded in paraffin. Eight-micrometer sections of the pericarp were stained using a saturated and filtered Sudan IV solution.

For scanning electron microscopy (SEM), pericarp samples were prepared from mature ER and WT fruit. The samples were fixed, dried using a critical point drier (LEICA EM CPD030; Leica, Germany) and coated with gold in a sputter coater (MC1000/ION SPUTTER; Hitachi, Japan), prior to observation using a field emission scanning electron microscope (SU8010; Hitachi, Japan) with an acceleration voltage of 15 kV as described by Yasuzumi et al. (1964).

### Water loss from ER fruit

For fruit water loss measurements, 15–20 ER and WT fruit were harvested from BC_5_F_1_ plants at 40 DAP and stored at room temperature. Three replicates were analyzed for each sample. The fresh weight of the fruits was measured at day 0 and every week, for 6 weeks. The water loss rate was calculated as the average percentage of weight loss (%), using the start weight values (Ws) and measured weight values (Wm) with the following formula: [(Ws−Wm)/Ws] × 100.

To identify fruit surface defects, a toluidine blue (TB) staining test was carried out using mature fruit, as described by Tanaka et al. (2004). Fruits from ER and WT plants were collected and immersed for 30 min in a 0.05% TB solution that had been filtered through a fiber media filter (0.2 mm).

### Fine mapping of the *ER4.1* gene

A previous study reported that *ER4.1* is located at the bottom of chromosome 4 (Monforte et al., 2001; Yeats et al., 2014). To map the location of the *ER4.1* gene, two heterozygous BC_5_ plants (N72-9, N72-5), which showed the ER phenotype, were selected, and segregating populations, derived from selfing progeny of the two plants, were used to select recombinant plants and for fine mapping of *ER4.1*. InDel (insertion-deletion) marker primers that identified polymorphisms between the ER and the WT plants were developed and used for mapping. The InDel loci were found by comparing the chromosome 4 sequence of LA0716 with that of *S. lycopersicum* Heinz 1706 in the Sol Genomics Network database (SGN, https://solgenomics.net/). All primers are shown in Table S11.

### RNA extraction and RNA-seq analysis

For the RNA-Seq analysis, to ensure consistency of the genetic background, homozygous plants derived from the segregation population described above were used. Fruit from the ER and WT plants were harvested at 15 DAP and at 23 DAP. Fruit peels were carefully scraped with a scalpel blade to minimize the removal of epidermal cells, and then rapidly frozen in liquid nitrogen and stored at −80°C until use. Two independent fruit peel samples were pooled for each sample. Total RNA was extracted using the EasyPure Plant RNA Kit (TransGen Biotech, China) and used for RNA-Seq library production. Briefly, mRNA was isolated from the total RNA samples using Oligo (dT) beads (Invitrogen). The mRNA was fragmented into short fragments and reverse-transcribed into cDNA using random primers. Sequencing adapters were ligated to the short fragments of each sample after purification and amplification. RNA-seq analysis was carried out using an Illumina Hiseq2000 (Berry Genomics Company). Three biological replicates for each sample were analyzed in parallel. The tomato reference genome sequence (SL2.50, http://solgenomics.net/) was used for read mapping, using Tophat software (Trapnell et al., 2009; Consortium, 2012).

Differentially expressed genes (DEGs) between ER and WT fruit were identified using a *p* < 0.001 in the R package DESeq tool (http://www.bioconductor.org/). Gene ontology (GO) analysis of the DEGs was performed using DAVID (The Database for Annotation, Visualization and Integrated Discovery, https://david.ncifcrf.gov/) and comparison with the most homologous *A. thaliana* genes.

### RT-PCR analysis

Total RNA was extracted from fruit peels of 15 and 23 DAP fruit (stages corresponding to the initial appearance of the ER phenotype) as described above, and then cDNA was synthesized using a reverse transcription kit (TransGen Biotech, China). Real-time qPCR analysis was performed using LightCycler 480 SYBR Green I Mastermix (Roche) on a LightCycler Roche 480 instrument, with default parameters. A tomato *ACTIN* gene (*Solyc03g078400*) was used as the reference gene and all analyses were performed using three technical and three biological replicates. All primers are listed in Table S11.

### Statistical analysis

All data were analyzed using SAS software. The measured values are presented as mean ± standard deviation (*SD*), and statistical significance of the differences between two samples was evaluated using a *t*-test for *P* = 0.05.

## Results

### Histological morphology of fruit pericarp tissue

**Figure 1:**
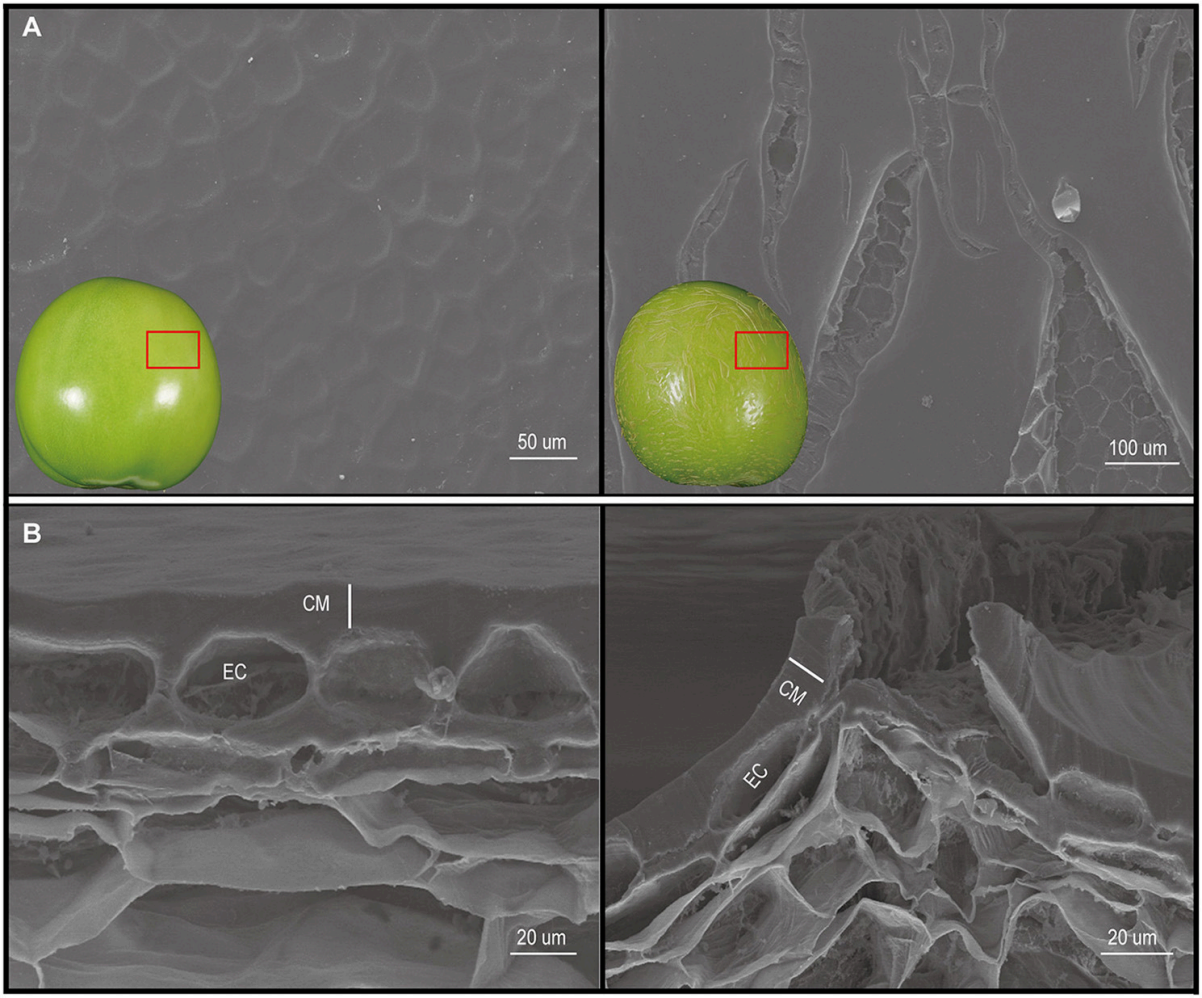
Scanning electron microscopy (SEM) observation of the cuticle of mature wild type (WT) and epidermal reticulation (ER) fruit. **(A)** Images of the fruit cuticle surface. **(B)** Images of fruit cuticle cross-sections. Images on the left are from the WT genotype, and those on the right are from the ER fruit. CM, cuticular membrane; EC, epidermal cell.

The fruit ER trait was derived from the introgression line IL4, which contains an introgression of a region from the bottom of *S. pennellii* (LA0716) chromosome 4 in the *S. lycopersicum* (1052) background. This trait has previously been found in other introgression lines that harbor the equivalent chromosomal segment from *S. habrochaites* acc. LA1777 (Yates et al., 2004). The cuticle of mature green fruit from the ER plants was reticulated and corky, with the appearance of a cantaloupe melon, whereas normal fruit have a smooth surface (Figure 1). In previous studies, it was shown that the ER phenotype was controlled by a single gene, *ER4.1*, in introgression line IL4-4 (Fulton et al., 2000).

To gain further insight into the ER cuticular morphology and microstructural changes, SEM and light microscopy were used to observe the histo-morphology of the fruit pericarp at different developmental stages. This revealed major differences between the cuticles of the ER and WT fruit; notably major fracturing of the ER cuticle (Figure 1), the extent of which correlated with the severity of the ER. Staining with Sudan IV dye revealed that the thickness of the outer cuticle increased gradually with fruit development (Figure S1), as has been reported for *S. lycopersicum* cv. M82 fruits (Buda et al., 2009). However, the thicknesses of the ER and normal fruit cuticles were similar at all the stages examined (Figure S1), suggesting that the reticulation in the ER fruit resulted from cuticle fracturing.

### Post-harvest water loss

**Figure 2:**
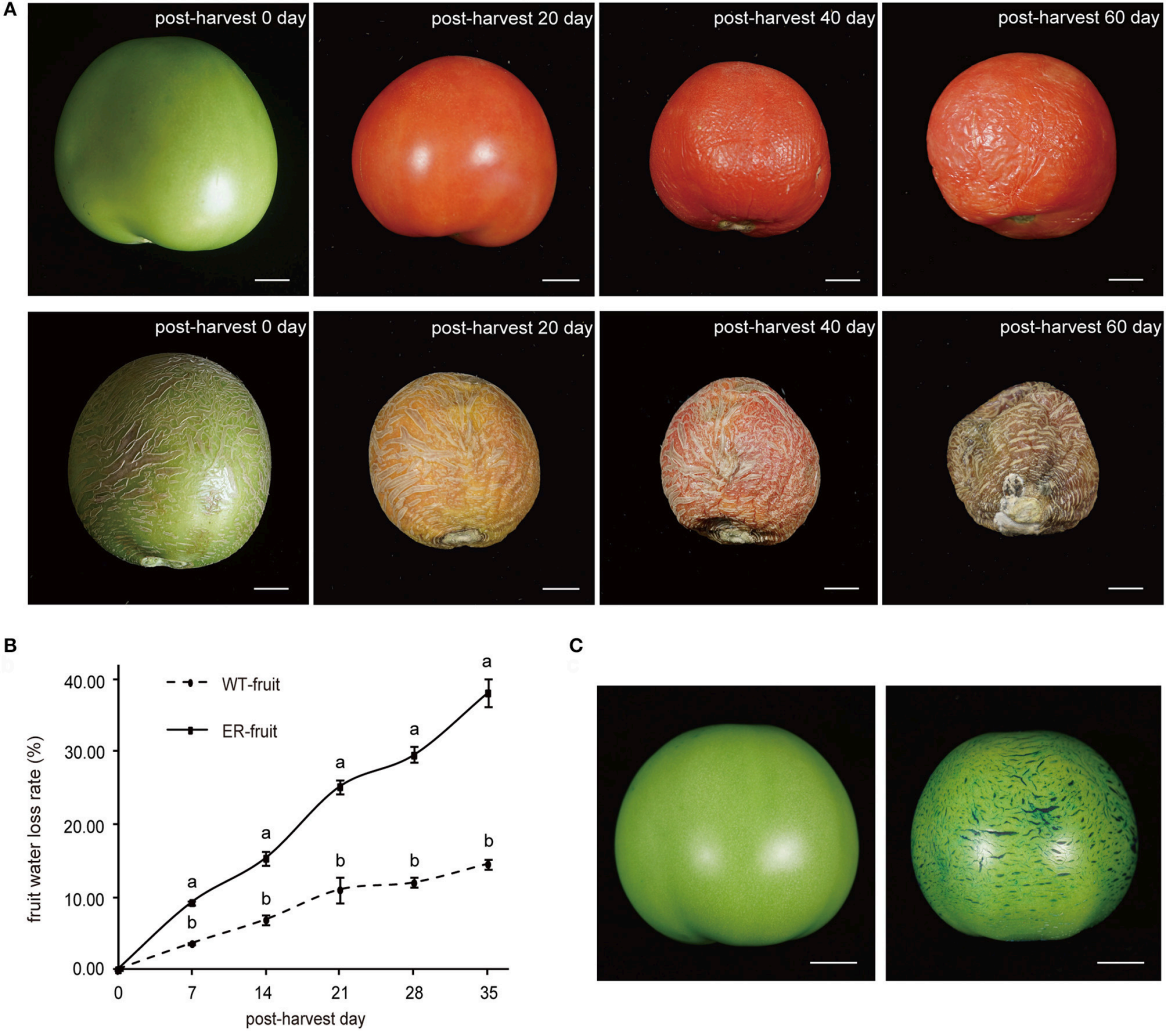
Analysis of transpirational water loss from mature wild type (WT) and epidermal reticulation (ER) fruit. **(A)** Comparison of the phenotypes of normal and ER fruits during postharvest storage. The upper row of fruit are WT and the lower row are ER. **(B)** Postharvest fruit water loss from WT and ER fruits over a 5-week period. The data are presented as the mean ± *SD*, and the different letters in the same days indicated significant difference between two samples at 0.05 level **(C)** Toluidine blue (TB, 0.5% solution) staining of WT (left) and ER (right) fruit 40 days after pollination (DAP). Scale bar = 1 cm.

The cuticle plays a vital role in protection against water loss from fruit tissues, and so to determine whether the ER fruit showed altered transpiration, we measured the weight change in ER and WT fruit stored at room temperature over a 5-week period (Figures 2A,B). ER fruit showed an approximately two-fold higher water loss rate than WT fruit within a few days of harvest (Figure 2B), and by 3 weeks were notably shriveled (Figure 2A). Mature green ER fruit that were submerged in TB for 30 min showed extensive staining in the reticulated areas (Figure 2C). We also observed that the ER fruit were vulnerable to microbial infection during storage. These results suggested that fruit cuticular defects were responsible for the ER phenotype. There were no observable differences in leaf phenotypes between the ER plants and WT plants (Figure S2).

### Fine mapping of *ER4.1*

**Figure 3:**
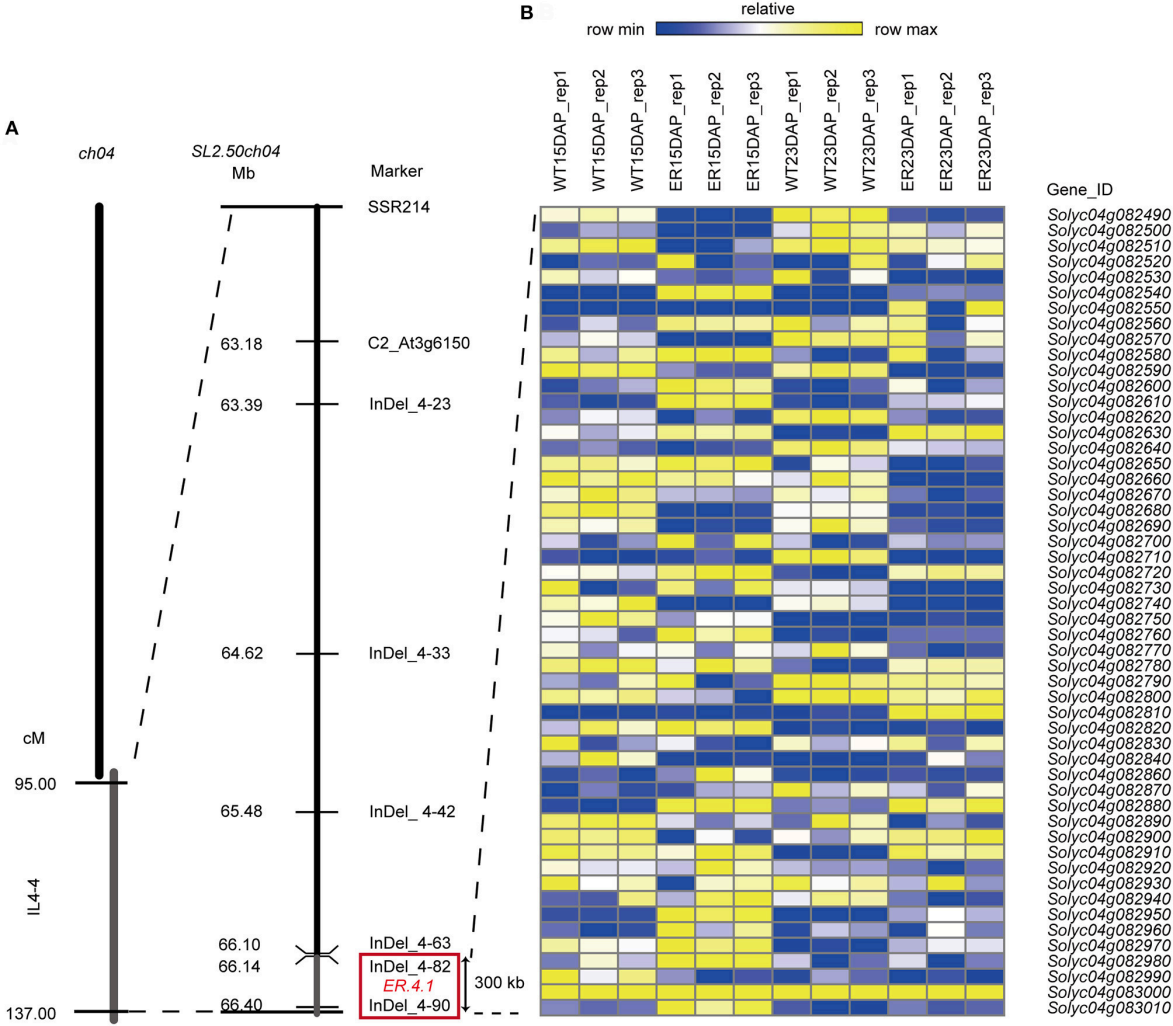
Fine mapping of *ER4.1* and analysis of candidate *ER4.1* genes using RNA-seq data. **(A)** Fine mapping of *ER4.1* using two BC_5_F_1_ and BC_5_F_2_ populations, mapped it to a ~300 kb region flanked by InDel 4–82 and InDel 4–90. **(B)** The relative expression levels of 52 genes predicted to be located in this region at 15 days after pollination (DAP) and 23 DAP in epidermal reticulation **(**ER) and WT fruit exocarp.

To examine the inheritance of the *ER4.1* gene, two hybrid plants, N72-5-23 and N72-5-40 (both backcross generation 5; BC_5_), showing an ER fruit phenotype, were selfed. The BC_5_S_1_ segregating population (309 plants) had a segregation ratio between ER-fruit and WT fruit phenotypes of ~3:1 [χ^2^ = 0.3158 and χ^2^ = 0.4777 < $\chi_{\left(0.05\right)}^{2}=$ 3.84; Table 1], indicating that the ER trait is controlled by a single dominant gene. This locus was initially mapped between two flanking markers (SSR214 and C2_At1g47830) to an ~42 Mb region of tomato chromosome 4 through map-based cloning. Line N72-5, N72-9, and 1052 have almost the same genetic backgrounds, other than the introgressed fragment of chromosome 4 from *S. pennellii*. This result was consistent with a previous report (Fulton et al., 2000; Yates et al., 2004). To further narrow the interval to *ER4.1*, 2,100, and 2,300 individuals from the BC_5_F_1_ and BC_5_F_2_ populations, respectively, were used for fine mapping, with 40 individuals displaying different recombination events in this region. Finally *ER4.1* was fine mapped to a ~300 kb region flanked by markers InDel 4-82 and InDel 4-90 (Figure 3A). According to the tomato genome annotation (ITAG2.40, https://solgenomics.net/), a total of 52 hypothetical genes are predicted to reside in this region (Table S1).

**Table 1:** χ2 test for the epidermal reticulation (ER) fruit trait of two independent segregating populations.

	**Line 5–23**		**Line 5–40**	
**Phenotype**	**Numbers**	**Probability**	**Numbers**	**Probability**
ER-fruit	111	0.7303	114	0.7261
Normal-fruit	41	0.2697	43	0.2739
total	152	1	157	1
χ^2^ value	0.3158		0.4777	
Probability of χ^2^	0.5741		0.4895	

*The segregation ratio fits 3:1, consistent with a single, dominant gene*.

### Comparative RNA-seq analysis of the ER and WT exocarp

As another approach to identifying *ER4.1* candidate genes, and to understand the molecular processes underlying the ER fruit skin phenotype, transcriptome analyses were performed to investigate gene expression changes in the ER fruit exocarp during development. WT fruits were used as control. RNA was extracted for RNA-seq analyses from the exocarp of fruit at two time points: 15 and 23 DAP (which corresponds to the initial appearance of the ER phenotype). Three biological replicates of all samples were analyzed using Illumina paired-end 125 bp sequencing. A total of 224,520,584 clean reads were generated, ~97% of which could be uniquely mapped to the ITAG2.40_cdna reference genome (Table S2). The expression of the genes was normalized using fragments per kilobase of transcript per million mapped reads (FPKM).

### Analysis of differentially expressed genes

**Figure 4:**
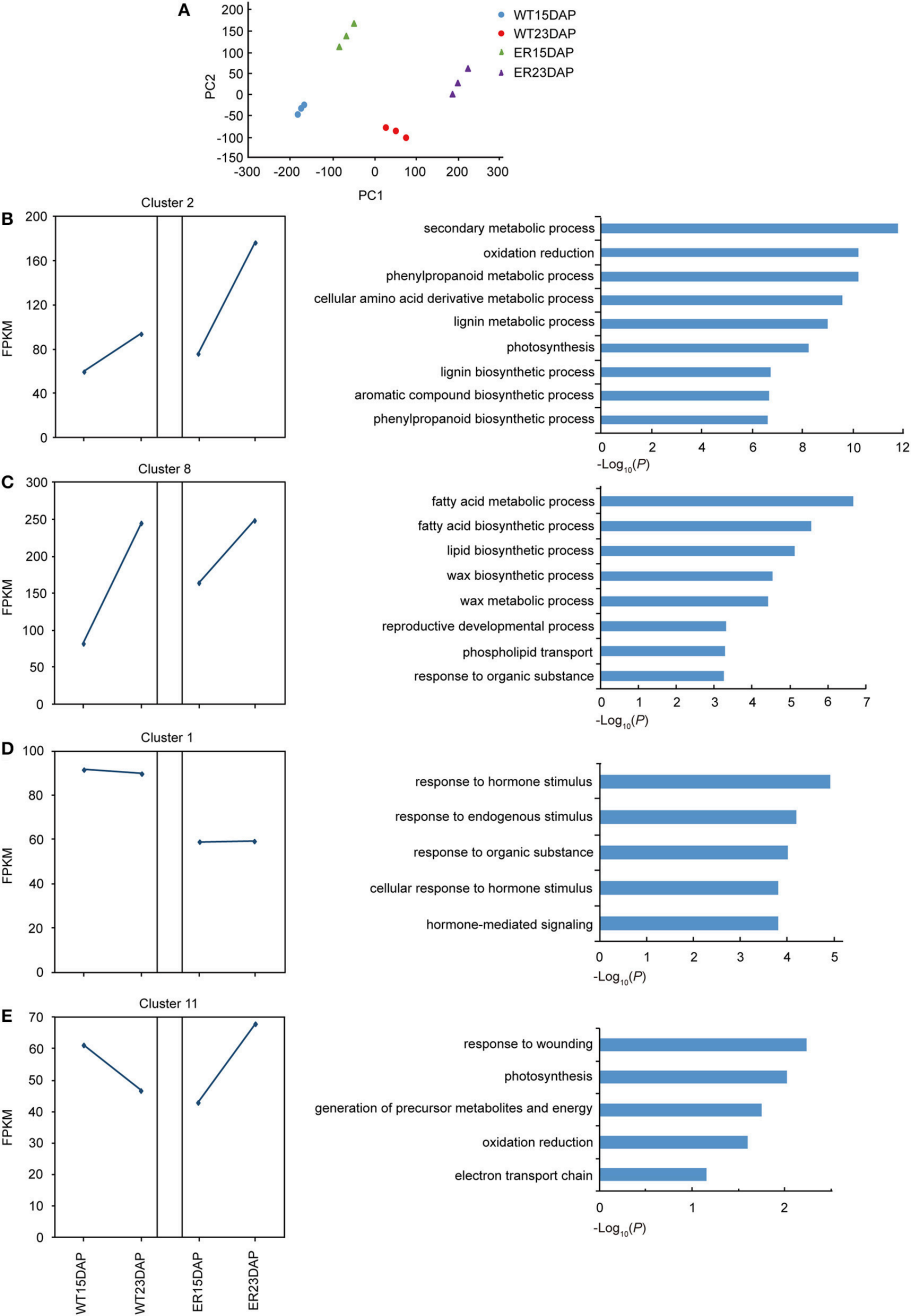
Identification of differentially expressed genes (DEGs) between epidermal reticulation (ER) and WT fruit exocarp samples. **(A)** Principle component analysis (PCA) of the RNA-seq datasets. The triangles indicate the ER samples and the circles indicate the WT samples. Green and purple indicate samples from 15 days after pollination (DAP) and 23 DAP in ER; blue and red indicate samples from 15 DAP and 23 DAP in WT, respectively. **(B–E)** The expression pattern (left) and the enriched GO terms in the “biological processes” category (right) of differentially expressed genes in cluster 2 **(B)**, 8 **(C)**, 1 **(D)**, and 11 **(E)**. The squares indicate the FPKM (fragments per kilobase of transcript per million mapped reads) value of the average of all clustered genes, and the blue lines show the expression pattern at the two time points in WT and ER fruit.

To assess the repeatability of the sequencing results, a principal component analysis (PCA) was performed and we observed that the three biological replicates co-clustered, and that four groups, corresponding to WT (15DAP), WT (23DAP), ER (15DAP), and ER (23DAP), were distinguishable (Figure 4A). The ER and CK fruit peel transcriptomes were both divergent at 15 and 23 DAP, which suggested that the expression profiles during ER fruit development were distinct from those in WT fruit.

A total of 4,478 DEGs between ER and WT peels were detected in at least one of the time points, using a rigorous *p*-value cut-off (< 0.01) (Table S3). At 15 and 23 DAP, 2,720 and 2,621 DEGs, respectively, were identified in the ER exocarp, compared with the WT fruit, and 863 DEGs were identified at both time points. A K-mean cluster analysis of gene expression patterns was performed and the DEGs were divided into 11 groups (Figure S3 and Table S4). Each group displayed a unique temporal expression pattern in ER and WT peel (Figure S4). To identify significantly altered biological processes associated with the DEGs in the ER fruit from the two stages, we carried out a Gene Ontology (GO) enrichment analysis. The DEGs were enriched not only in processes involving cuticle biosynthesis, but also significantly in the “response to hormone,” “photosynthesis,” “stress response,” and other categories (Table S5). The largest cluster was Cluster 2, which contained genes that did not show a higher expression in the ER samples than in the WT samples at 15 DAP, but had a higher expression at 23 DAP (Figure 4B). Genes in this cluster were enriched in the “phenylpropanoid” and “lignin metabolism” categories (Figure 4B). In Cluster 8, genes in the ER exocarp at 15 DAP were expressed at distinctly higher levels than in WT, but at 23 DAP they had a similar level of expression to WT. This cluster was enriched in genes from the “response to fatty acid,” “lipid and wax” metabolic processes (Figure 4C), which is consistent with the idea that cuticle biologybeing affected to some degree by *ER4.1* gene regulation. In contrast with these two clusters, Cluster 1 included genes that showed a lower expression level in the ER samples at 15 and 23 DAP than in the WT samples (Figure 4D). GO terms enriched in this cluster were mainly “response to hormone stimulus” and “stress” (Figure 4D). The expression levels of genes in Cluster 11 were lower in 15 DAP ER fruit, and then higher in 23 DAP fruit compared to WT peel (Figure 4E). GO enrichment analysis indicated that the genes in this cluster were enriched in the “response to wounding” category (Figure 4E). Taken together, these results suggest that the expression of defense-responsive genes altered in 23 DAP ER fruit compared with similarly staged WT fruit. Finally, some of the DEGs in the “biological processes” category were also associated with the specialized metabolism of cuticle formation (flavonoids) and polysaccharides (Table S5).

To validate the RNA-seq analysis results, we selected several DEGs related to the cuticle and performed RT-PCR analyses. The RT-PCR results were consistent with the RNA-seq data (Figure S5).

### Analysis of candidate *ER4.1* genes using RNA-seq

To identify the best *ER4.1* candidate genes, we further analyzed the expression dynamics of the 52 hypothetical genes from the fine mapping interval in the two developmental stages of ER and WT exocarp using the RNA-seq data. We identified 20 DEGs in this region using a rigorous *p*-value cut-off (< 0.01). Of these, 4 and 8 genes were detected in the 15 and 23 DAP ER fruit exocarp samples, respectively. We also identified 8 genes that were significantly differently expressed at both 15 and 23 DAP (Figure 3B and Table S1). For example, *Solyc04g082540* was expressed at significantly higher levels at both time points (*p* = 5.73E-307, *p* = 7.69E-193, respectively) in the ER fruit peel than in WT. *Solyc04g082540* is predicted to encode an protein of unknown function, and has previously been reported as the *CUTICULAR WATER PERMEABILITY 1* (*CWP1*) gene. The expression of this gene as an introgression into *S. habrochaites* was shown to increase the water permeability of the fruit cuticle, but not to alter the composition of waxesorcutin monomers (Hovav et al., 2007). *Solyc04g082950* encodes a protein of unknown function containing a DUF23 domain, which is annotated as belonging to glycosyltransferase family 92 in the PFAM database (*p* = 3.36E-28, *p* = 2.29E-30). The expression of this gene was also substantially up-regulated in the ER peel compared to WT. *Solyc04g082630* and *Solyc04g082910* were expressed at higher levels at 23 DAP (*p* = 9.16E-15 and *p* = 3.17E-15, respectively) in ER fruit than in WT, and are predicted to encode a glyceraldehyde-3-phosphate dehydrogenase B and a protein of unknown function containing a CP12 domain, respectively. The latter may be involved in carbohydrate metabolism in the Calvin cycle (Wedel and Soll, 1998). Other DEGs that were expressed at higher or lower levels in the ER samples than in WT fruit are shown in Figure 3B and Table S1.

### Analysis of genes related to cuticle biosynthesis

The expression of several genes associated with cuticle biosynthesis (e.g., cutin and wax biosynthesis and assembly) were notably different in the ER peel compared to the WT (Tables S6, S7). Examples of such genes involved in cutin biosynthesis included: *LACS4* (*Solyc01g095750*), a long-chain acyl CoA synthetase putatively involved in the acyl activation of fatty acids and very-long-chain-fatty acid (VLCFA) biosynthesis (Jessen et al., 2011); four CYP450 genes of which *CYP77A1* (*Solyc11g007540*) and *CYP77A2* (*Solyc05g055400*) are known to be responsible for the midchain hydroxylation of C16 fatty acids (Li-Beisson et al., 2009), while *CYP86A7* (*Solyc08g081220*) and *CYP86A68* (*Solyc01g094750*) catalyze terminal hydroxylation reactions during cutin biosynthesis (Shi et al., 2013); and *HTH, H2H like1*, and *HtH like2* genes (*Solyc00g156980, Solyc08g080190*, and *Solyc06g035580*), which are putatively involved in the formation of dicarboxylic fatty acids (DFA) (Kurdyukov et al., 2006b). The genes involved in cutin monomer synthesis were only up-regulated at 15 DAP in ER fruit, and then maintained a similar level of expression to that in the WT sample at 23 DAP (Table S6). The genes involved in extracellular transport and polymerization of cutin, ABCG32 (*Solyc06g065670*) and GDSL-lipase genes (*Solyc04g081770* and *Solyc07g049440*), also showed a higher expression level in the ER peel at 15 DAP than in the WT peel (Table S6).

**Figure 5:**
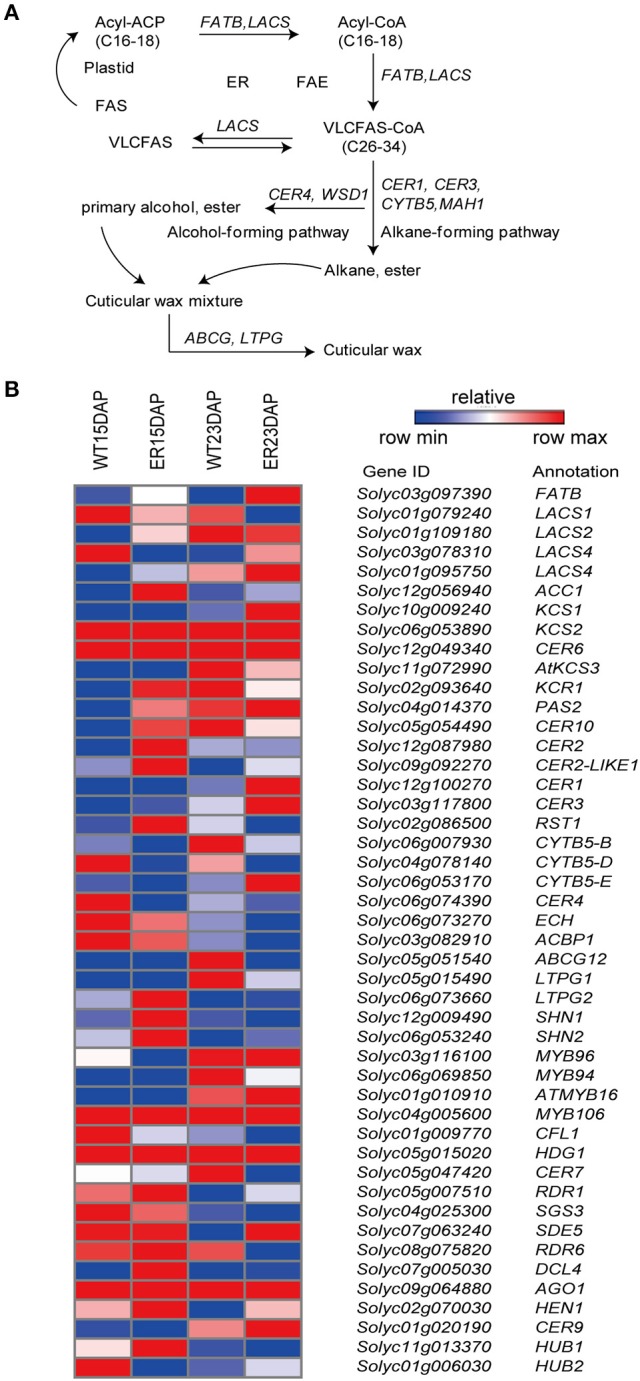
Analysis of differentially expressed genes (DEGs) involved in the wax biosynthesis pathway between epidermal reticulation (ER) and WT fruit exocarp. **(A)** Schematic diagram of wax biosynthesis (Saetbuyl and Michung, 2015). **(B)** The expression profile of genes encoding key enzymes in the pathway. The FPKM (fragments per kilobase of transcript per million mapped reads) value is the average of three biological replicates. Abbreviations are listed in Table S12.

Several wax-related genes were identified as being differentially expressed between the ER and WT exocarp (Figure 5 and Table S7). These included: *ACC1 (Solyc12g056940)*, an acetyl-CoA carboxylase that is required for malonyl-CoA elongation reactions (Lü et al., 2011); *CER6* (*Solyc02g085870*), which has been reported to be involved in the synthesis of VLCFA precursors for the production of cuticular waxes (Hooker et al., 2002); a BAHD acyltransferase, *CER2* (*Solyc12g087980*), which is putatively required for extension of VLCFAs to C30 in *A. thaliana* (Haslam et al., 2012); and *CER3* (*Solyc03g117800*), which has been reported to contribute to the synthesis of alkanes, the most abundant components of cuticular waxes in tomato (Bernard et al., 2012). These genes were all more highly expressed at 15 DAP in the ER fruit peel than in WT. In contrast, the *CER4/FAR3* (*Solyc06g074390*) gene, encoding an alcohol-forming fatty acyl-CoA reductase (FAR), which is required for the synthesis of primary alcohols, was expressed at lower levels at this time point (Rowland et al., 2006). In addition, two tomato oxidosqualene cyclase (OSC) genes, *SlTTS1* and *SlTTS2* (*Solyc12g006520* and *Solyc12g006530*), which are involved in triterpenoid biosynthesis (Wang et al., 2011), showed a higher expression at 15 DAP in the ER fruits than in the WT. Taken together, these results suggested that the expression level of the genes related to many steps in cuticle biosynthesis are abnormal in ER fruit.

### Analysis of genes related to flavonoid biosynthesis

**Figure 6:**
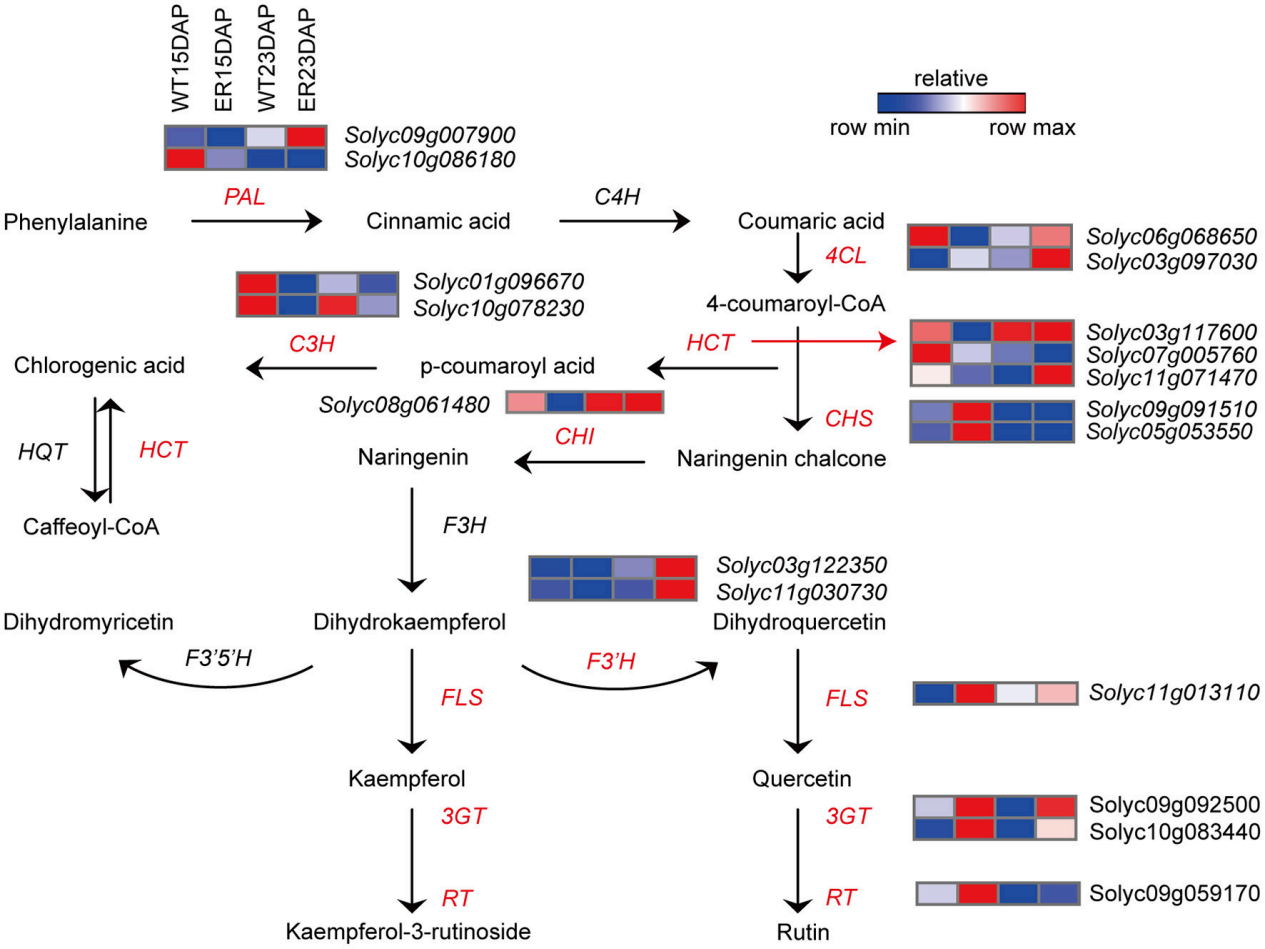
Analysis of differentially expressed genes (DEGs) involved in the flavonoid biosynthesis pathway between epidermal reticulation (ER) and WT fruit exocarp. Schematic diagram of the flavonoid biosynthesis (Ballester et al., 2010) and the expression profile of DEGs encoding key enzymes in the pathway. The FPKM (fragments per kilobase of transcript per million mapped reads) value is the average of three biological replicates. Abbreviations are listed in Table S12.

We also observed that the expression of several genes involved in phenylpropanoid and flavonoid biosynthesis were distinctly different in the ER fruit peel compared to the WT sample (Figure 6 and Table S8). The expression levels of genes associated with several key steps in the flavonoid biosynthetic pathway, including genes encoding phenylalanine ammonia-lyase (PAL), 4-coumarate: CoA ligase (4CL), chalcone isomerase (CHI), flavonoid-3′-hydroxylase (F3′H), cinnamoyl-CoA shikimate/quinate transferase (HCT) and *p*-coumaroyl ester 3-hydroxylase (C3H), were significantly lower (by almost two-fold) in 15 DAP ER fruit exocarp compared to WT fruit. In contrast, the expression levels of genes encoding chalcone synthase (CHS), flavanone-3-hydrolase (F3H), flavonol synthase (FLS), flavonoid-3-O-glucosyltransferase (3GT), and flavonoid 3-O-glucoside-rhamnosyltransferase (RT) were significantly higher at this time point compared to WT (Bovy et al., 2002; Ballester et al., 2010; Ambawat et al., 2013). Interestingly, genes involved in these pathways were rarely differently expressed between ER and WT fruits at 23 DAP (Figure 6 and Table S8).

Besides the cuticle-associated pathways, several cell wall and hormone pathway related genes also showed differences in expression between ER and WT (Table S9), suggesting that the gene underlying the ER phenotype has pleiotropic effects.

### Analysis of transcription factors (TFs) related to cuticle biosynthesis

We next examined changes in the expression of transcription factors (TFs) based on the RNA-seq data. We identified 202 differentially expressed TFs, belonging to 35 families, including the AP2 (4), bHLH (25), C2H2 zinc finger (14), HD-ZIP (14), MYB (19), WRKY (20), and other TF categories (106) (Table S10). Most of these families have previously been associated with the fruit epidermis/cuticle. For example, four genes from the AP2 TF family, of which several members have been found to regulate cuticle related genes (Mintz-Oron et al., 2008; Shi et al., 2013), were expressed at lower levels in ER than in the WT sample at 15 DAP (Table S10). Twelve genes encoding MYB TFs showed a lower level of expression at 15 DAP in ER compared to WT, while only 2 showed higher expression. At 23 DAP, 7 and 4 showed increased and decreased expression, respectively (Table S10). MYB TFs are components of regulatory networks controlling development, metabolism, and responses to biotic and abiotic stresses (Ambawat et al., 2013). They are believed to be involved in regulating the flavonoid pathway in tomato fruit, as well as the synthesis of cuticular waxes (Ballester et al., 2010). The differently expressed HD-ZIP TF genes were also expressed at much lower levels in the ER sample than in WT at 23 DAP (Table S10). HD-ZIP family members have been reported to play a key role in epidermal cell differentiation, anthocyanin accumulation, the regulation of lipid biosynthesis and transport, and cuticle biosynthesis (Isaacson et al., 2009; Nadakuduti et al., 2012). In addition, we identified many differentially expressed TFs belonging to the WRKY, ARF, ERF, and NAC families (Table S10).

## Discussion

Tomato is one of the most popular consumed vegetable crops worldwide, and fruit cracking can cause serious economic losses and poor marketability (Peet, 1992). Fruit cracking is a physiological disorder associated with fractures in the fruit cuticle, or splitting as an extreme form of cracking that penetrates into the flesh (Opara, 1996).

In this study, we describe a tomato fruit phenotype that we refer to as epidermal reticulation (ER) (Figure 2A) among to the cuticle cracking, and fruits with a corky, reticulated epidermis on the mature fruit skin that resembles a “melon-like” fruit skin. The phenotype was shown to be controlled by a dominant gene derived from the introgression of an allele from a wild tomato species *S. pennellii* (LA0716). Fine mapping of *ER4.1* identified a ~300 kb region on chromosome 4 between InDel 4-82 and InDel 4-90 (Figure 3A) containing a total of 52 hypothetical genes, of which 20 were differentially expressed between the ER and WT genotypes, based on RNA-seq data (Figure 3B and Table S1). Among these genes, a predicted transcription factor was not found based on sequence annotation. Four candidate genes were predicted by the gene expression abundance in FPKM and annotation (Table S1). One of the four candidate genes, *Solyc04g082540* (*CWP1*) encodes a protein of unidentified function in theDUF833 domain family, which has previously been reported to cause cuticle microcracking in tomato fruit (Hovav et al., 2007). However, we found that the fruit phenotype in our study was not exactly the same as the previously reported *cwp1* mutant. Specifically, the ER fruit showed reticulated cracking that was evident by visual examination, which was more severe *cwp1*. This is probably because the allelic form of the gene in our introgression line population was derived from different wild species than that reported by Hovav et al. (2007). *Solyc04g082950, Solyc04g082630*, and *Solyc04g082910* may be involved in carbohydrate metabolism in the Calvin cycle. A previous study showed that an increase in assimilate supply to tomato fruit may promote cuticle crack by influencing water flow to the fruit, thereby affecting turgor pressure (Guichard et al., 2001). Thus, *CWP1, Solyc04g082950, Solyc04g082630*, and *Solyc04g082910* represent potential candidate genes for the *ER4.1* locus, although we note that there are many genes in the fine-mapping region. One approach to define the *ER4.1* locus would be to create a larger population to isolate additional recombinants; however, we are in the process of generating transgenic tomato lines with altered expression of each of the four candidate genes to assess their potential association with the ER phenotype.

In tomato fruit, cutin and waxes are key factors in limiting water loss and providing physical protection, as is the case with *A. thaliana* (Bargel and Neinhuis, 2005). For example, the fruits of the *S. lycopersicum eceriferum6 (lecer6)* mutant show increased transpirational water loss and wrinkling of the fruit surface during ripening due to the lack of C31 alkanes, although the total wax content is similar to that in WT fruit (Leide et al., 2007). Moreover, the *delayed fruit deterioration* (*dfd*) mutant has a higher total wax and cutin content of the fruit than WT, resulting in increased susceptibility to water loss (Saladié et al., 2007). Isaacson et al. (2009) reported that fruit of the tomato mutant *cutin deficient 2 (cd2*) had an altered water loss; however, the amount of cutin did not correlate with the degree of water loss. In our study, no difference in fruit cuticle thickness was found between ER and WT fruit (Figure S1) and while a portion of the water loss likely occurred through the fruit pedicel scar, the ER fruits showed higher water loss rates than equivalent sized WT fruit. Thus, we suggested that the water loss in ER fruit was substantially higher because of the fissuring and physical discontinuities of the cuticle (Figure 1).

To better understand the mechanism of ER formation in tomato fruit, we examined the transcriptome dynamics and differential expression of genes in ER and WT exocarp using RNA-seq analysis. The results suggested that the synthesis and modification of compounds associated with the cuticle (cutin and waxes) was associated with the phenotype, and that metabolism of phenylpropanoids, and specifically flavonoids, was substantially altered in the exocarp transcriptome of ER fruit. The *CER3* (*Solyc03g117800*) gene, which is located in the alkane-forming pathway of tomato cuticular waxes, was more highly expressed at 15 DAP in ER fruit peel than in WT, while the *CER4/FAR3* (*Solyc06g074390*) gene from the primary alcohol pathway was expressed at lower levels (Bernard and Joubès, 2013). The *SlTTS1*and *SlTTS2* (*Solyc12g006520* and *Solyc12g006530*) genes involved in triterpenoid biosynthesis were expressed at higher levels in ER plants than in WT plants (Figure 5 and Table S7). The accumulation of flavonoids may be affected in the ER fruit since many of the genes involved in the flavonoid metabolic pathways were differentially expressed between ER and WT fruit (Figure 6 and Table S8). Naringenin accumulation is regulated by *CHS*, which was expressed at high levels in ER fruit (Figure 6 and Table S8), and may contribute to peroxidation of the cuticle (Andrews et al., 2002). España et al. (2014) showed, by means of silencing *CHS* expression, that flavonoid accumulation may be related to water transpiration water loss. In the *y* and *dfd* mutants, an absence of flavonoids from the fruit epidermis has been reported not to correlate with reduced cuticle formation (Saladié et al., 2007; España et al., 2014). The most notable ER trait was fruit cuticle cracking and while the metabolic function of *ER4.1*gene has yet to be definitively demonstrated, our results suggested the cuticle may be affected directly, or indirectly, by the expression of *ER4.1*.

The fracture mechanics of the fruit skin are complex and previous studies have suggested fracturing is caused by the biochemical composition of the cuticle. Indeed, fruit size and shape, fruit development, sugar synthesis, and transport as well as environmental conditions may all influence fruit cracking (Dorais et al., 2004). Alternatively, the cuticle fissuring may be caused by some currently undetermined compounds during cuticle assembly, or by interactions between the epidermis and the underlying tissues, rather than to the cuticle *per se*. While the mechanism of fruit ER formation remains to be determined, our study provides new insights and will help guide future studies of the relationship between *ER4.1* expression and epidermal reticulation.

## Author contributions

XW conceived of and designed the study. LC and ZQ performed most of the experiments with help from ZW, ZH, JG, YG, and YD. XW, LC, and ZQ wrote the paper.

### Conflict of interest statement

The authors declare that the research was conducted in the absence of any commercial or financial relationships that could be construed as a potential conflict of interest.
