# Supplementary material for: Fine Mapping of a Gene (ER4.1) that Causes Epidermal Reticulation of Tomato Fruit and Characterization of the Associated Transcriptome
Source: Front Plant Sci. 2017 Jul 26;8:1254. doi: 10.3389/fpls.2017.01254 (PMC5526902; doi:10.3389/fpls.2017.01254)
Supplement: Supplementary file 2 [file Table2.doc]

| Sample | Total raw reads | Total unique  mapped reads | Total unique  mapped reads % |
| --- | --- | --- | --- |
| WT15DAP_rep1 14,331,387 97.53% | 14,693,638 | 14,331,387 | 97.53% |
| WT15DAP_rep2 | 15,963,244 | 15,552,493 | 97.43% |
| WT15DAP_rep3 | 17,534,529 | 17,086,469 | 97.44% |
| WT23DAP_rep1 | 19,141,624 | 18,672,671 | 97.55% |
| WT23DAP_rep2 | 14,627,139 | 14,255,604 | 97.46% |
| WT23DAP_rep3 | 16,845,442 | 16,418,555 | 97.47% |
| ER15DAP_rep1 | 16,372,488 | 15,902,843 | 97.13% |
| ER15DAP_rep2 | 15,532,935 | 15,163,906 | 97.62% |
| ER15DAP_rep3 | 17,960,985 | 17,695,218 | 98.52% |
| ER23DAP_rep1 | 33,622,400 | 32,514,234 | 96.70% |
| ER23DAP_rep2 | 15,381,164 | 15,022,007 | 97.66% |
| ER23DAP_rep3 | 26,844,996 | 26,019,572 | 96.93% |
| Total raw reads | 224,520,584 | 218,634,959 | 97.38% |

**Supplementary Table S2. Overview of RNA-seq data from ER and WT fruit exocarp at 15DAP and 23 DAP.**
