# Supplementary material for: Fine Mapping of a Gene (ER4.1) that Causes Epidermal Reticulation of Tomato Fruit and Characterization of the Associated Transcriptome
Source: Front Plant Sci. 2017 Jul 26;8:1254. doi: 10.3389/fpls.2017.01254 (PMC5526902; doi:10.3389/fpls.2017.01254)
Supplement: Supplementary file 12 [file Table12.doc]

**Supplementary Table S12. The full name of abbreviations presented in this paper.**

Abbreviations

ABCG ATP binding cassette (ABC) transporter

ACBP Acyl-CoA binding protein

Acyl-ACP Acyl–acyl carrier protein

CER Eceriferum

CYTB5 Cytochrome b5 isoform

ER Endoplasmic reticulum

FAE Fatty acids elongase

FATB Fatty acyl-ACP thioesterase

LACS Long chain acylcoenzyme A synthetase

LTP Lipid transfer protein

LTPG Glycosylphosphatidylinositol-anchored LTP

MAH Midchain alkane hydroxylase

VLCFA Very long chain fatty acid

WSD Bifunctional wax synthase/acyl-CoA:diacylglycerol acyltransferase

PAL Phe ammonia-lyase

C4H Cinnamate 4-hydroxylase

4CL 4-coumarate:CoA ligase

HCT Cinnamoyl-CoA shikimate/quinate transferase

C3H P-coumaroyl ester 3-hydroxylase

HQT Hydroxycinnamoyl-CoA quinate transferase

CHS Chalcone synthase

CHI Chalcone isomerase

F3H Flavanone-3-hydroxylase

F3’H Flavonoid-3’-hydroxylase

F3’5’H Flavonoid-3’5’-hydroxylase

FLS Flavonol synthase

3GT Flavonoid-3-O-glucosyltransferase

RT Flavonoid 3-O-glucoside-rhamnosyltransferase
