# Supplementary figures and images for: Fine Mapping of a Gene (ER4.1) that Causes Epidermal Reticulation of Tomato Fruit and Characterization of the Associated Transcriptome
Source: Front Plant Sci. 2017 Jul 26;8:1254. doi: 10.3389/fpls.2017.01254 (PMC5526902; doi:10.3389/fpls.2017.01254)

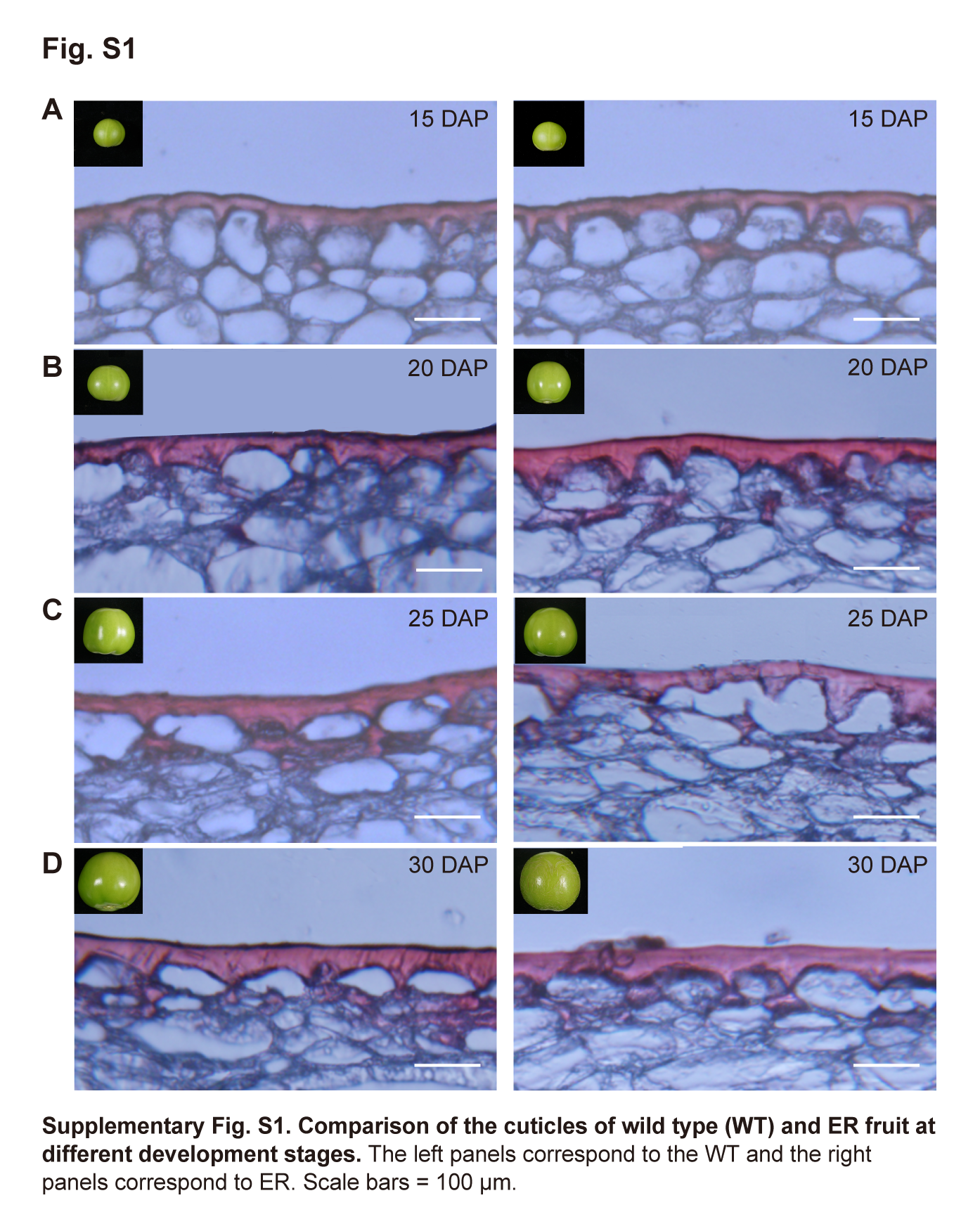

Supplement: Supplementary file 13 [file Image1.TIF]

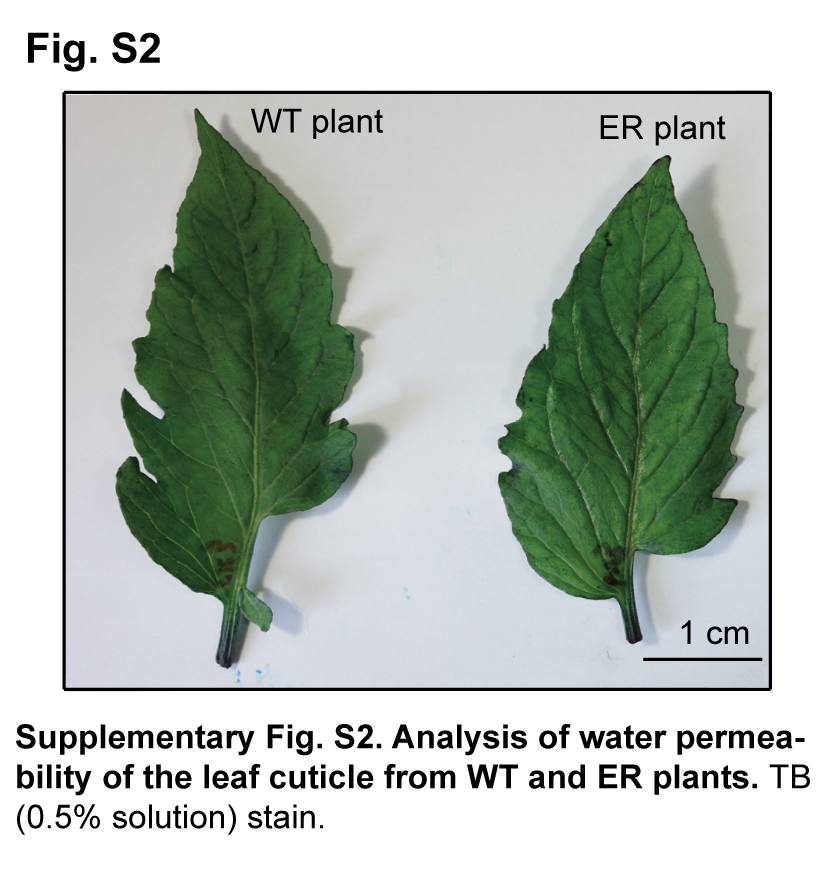

Supplement: Supplementary file 14 [file Image2.TIF]

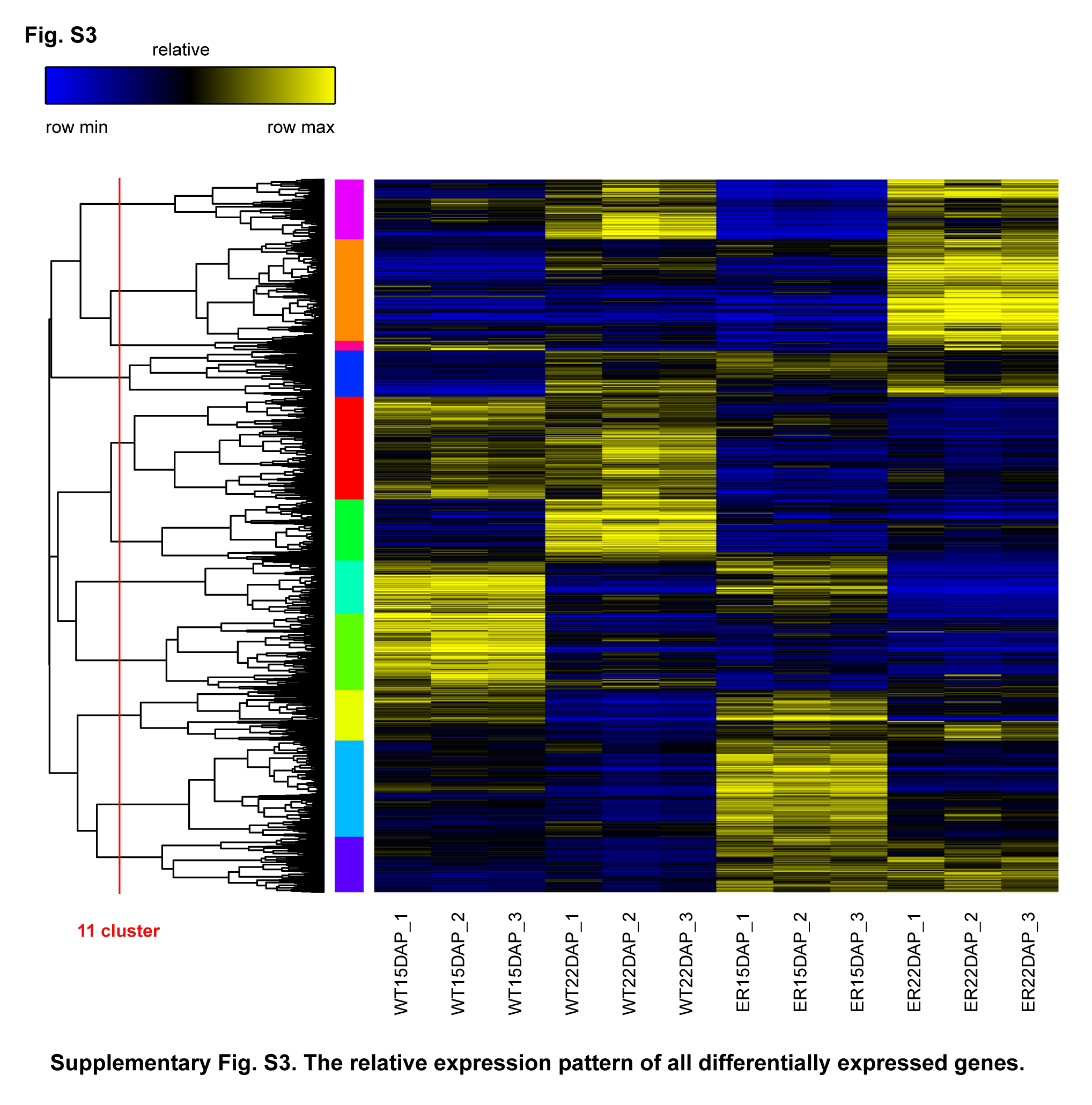

Supplement: Supplementary file 15 [file Image3.TIF]

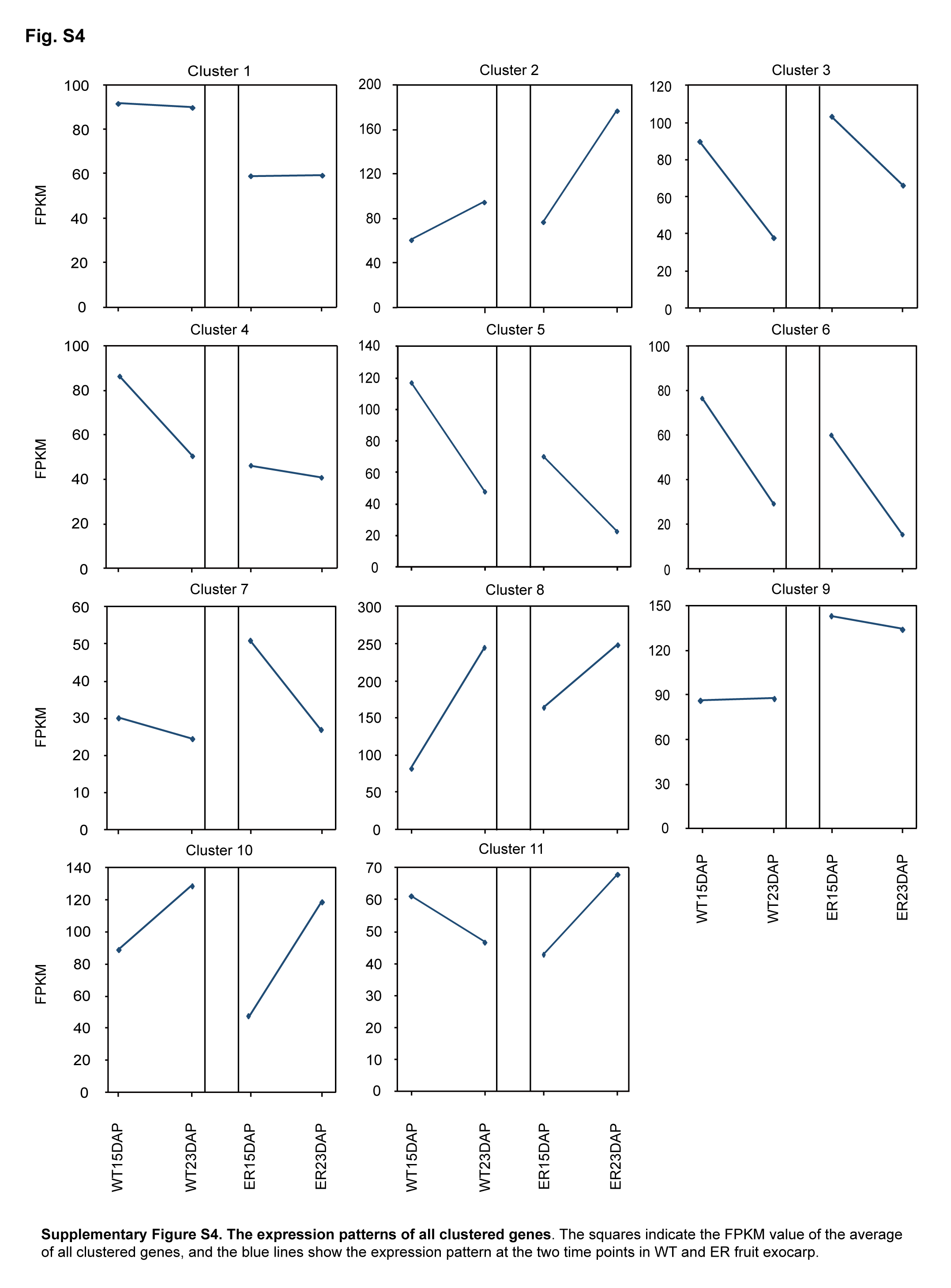

Supplement: Supplementary file 16 [file Image4.TIF]

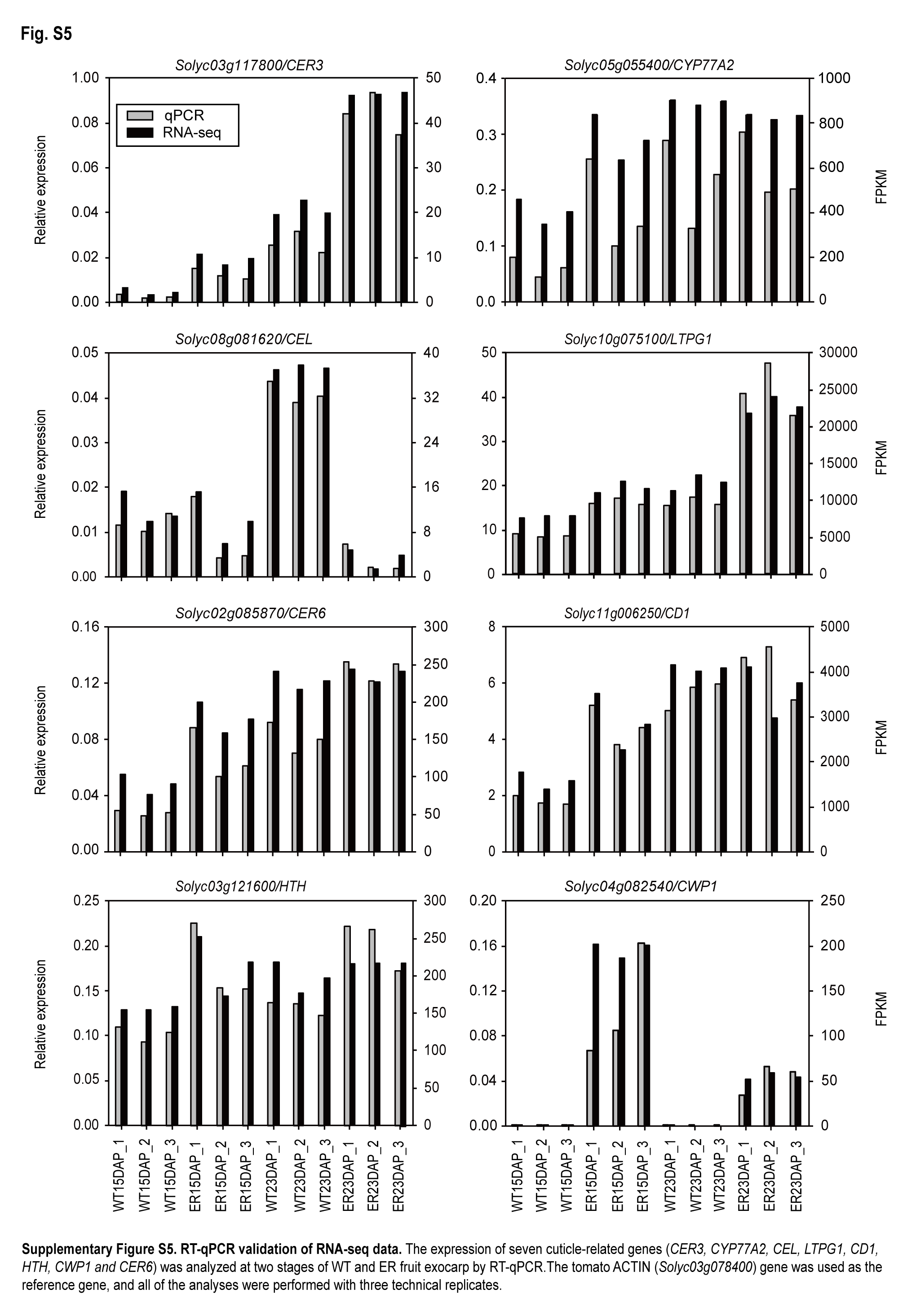

Supplement: Supplementary file 17 [file Image5.TIF]
